# Intraspecies and interspecies transmission of mink H9N2 influenza virus

**DOI:** 10.1038/s41598-017-07879-1

**Published:** 2017-08-07

**Authors:** Zhao Yong-feng, Diao Fei-fei, Yu Jia-yu, Zhang Feng-xia, Jiang Chang-qing, Wang Jian-li, Guo Shou-yu, Cui Kai, Liu Chuan-yi, Wei Xue-hua, Shi-jin Jiang, Xie Zhi-jing

**Affiliations:** 1 Shandong Provincial Key Laboratory of Animal Biotechnology and Disease Control and Prevention, Taian, Shandong 271018 China; 2 College of Veterinary Medicine, Shandong Agricultural University, Taian, Shandong 271018 China; 3 College of Animal Science and Veterinary Medicine, Qingdao Agricultural University, Qingdao, Shandong 266109 China

## Abstract

H9N2 influenza A virus (IAV) causes low pathogenic respiratory disease and infects a wide range of hosts. In this study, six IAVs were isolated from mink and identified as H9N2 IAV. Sequence analysis revealed that the six isolates continued to evolve, and their PB2 genes shared high nucleotide sequence identity with H7N9 IAV. The six isolates contained an amino acid motif PSRSSR↓GL at the hemagglutinin cleavage site, which is a characteristic of low pathogenic influenza viruses. A serosurvey demonstrated that H9N2 IAV had spread widely in mink and was prevalent in foxes and raccoon dogs. Transmission experiments showed that close contact between H9N2-infected mink and naive mink, foxes and raccoon dogs resulted in spread of the virus to the contact animals. Furthermore, H9N2 challenge experiments in foxes and raccoon dogs showed that H9N2 IAV could infect these hosts. Virological and epidemiological surveillance of H9N2 IAV should be strengthened for the fur animal industry.

## Introduction

Mink are known to be susceptible to IAVs. Since 1984, several IAV subtypes, such as H10N4, H3N2, swH3N2/pH1N1, H1N2 and H9N2^1–7^, have been isolated from mink. Both SAα2,3-Gal and SAα2,6-Gal were detected in the respiratory track of mink^5^, therefore, mink could serve as intermediary influenza virus hosts between poultry and humans. Two H5N1 IAVs were isolated from raccoon dogs that died with respiratory disease in China^8^. It has been reported that red foxes fed bird carcasses infected with H5N1 IAV could excrete virus while remaining free of severe disease, thereby potentially playing a role in virus dispersal^9^.

H9N2 IAVs are currently widespread in wild birds, poultry and mammals in Asia and have caused a few cases of influenza in humans^10, 11^. H9N2 IAV eradication is not a priority for animal disease control in many countries, and H9N2 IAVs continue to evolve and spread^12–14^. H9N2 IAVs were likely to have facilitated the evolution of H7N9 in China^15–17^. In 2013, H9N2 IAVs were isolated from mink in Shandong, and the seroprevalence of antibodies to H9 in mink was 20%, suggesting that H9N2 IAVs are prevalent in mink^5^. In some areas in China, mink, foxes and raccoon dogs are raised on the same farms, which could increase the chance for H9N2 IAV to cross the species barrier. In this study, we analyzed the biological characteristics and variation of H9N2 IAVs in mink. A serosurvey for anti-H9N2 antibody in mink, foxes and raccoon dogs was performed to demonstrate whether anti-H9 antibodies were widespread in these hosts. Animal experiments were carried out to clarify whether close contact between experimentally H9N2 infected mink and naive mink, foxes and raccoon dogs could lead to intraspecies and interspecies transmission, and whether experimental intranasal infection of foxes and raccoon dogs with mink H9N2 IAV resulted in virus shedding, clinical signs and pathological lesions.

## Result

### Virus isolation and serosurvey

In this study, six IAVs were isolated from mink, named as A/Mink/Shandong/Z1/2015, A/Mink/Shandong/Z2/2015, A/Mink/Shandong/Z3/2015, A/Mink/Shandong/Z4/2015, A/Mink/Shandong/Z5/2015 and A/Mink/Shandong/Z6/2015. The six isolates were identified as H9N2 IAV by RT-PCR. However, attempts at IAV isolation from foxes and raccoon dogs were unsuccessful. 97 of the 313 (31.0%) serum samples from mink were positive for anti-H9 antibody and the HI titers were 16–1024. 76 of 128 (59.4%) serum samples from foxes were positive and the HI titers were 16–2048. 106 of 256 (41.4%) serum samples from raccoon dogs were positive and the HI titers ranged from 16 to 64. The serum samples were negative for anti-H1 antibody.

### Genetic analysis

**Figure 1:**
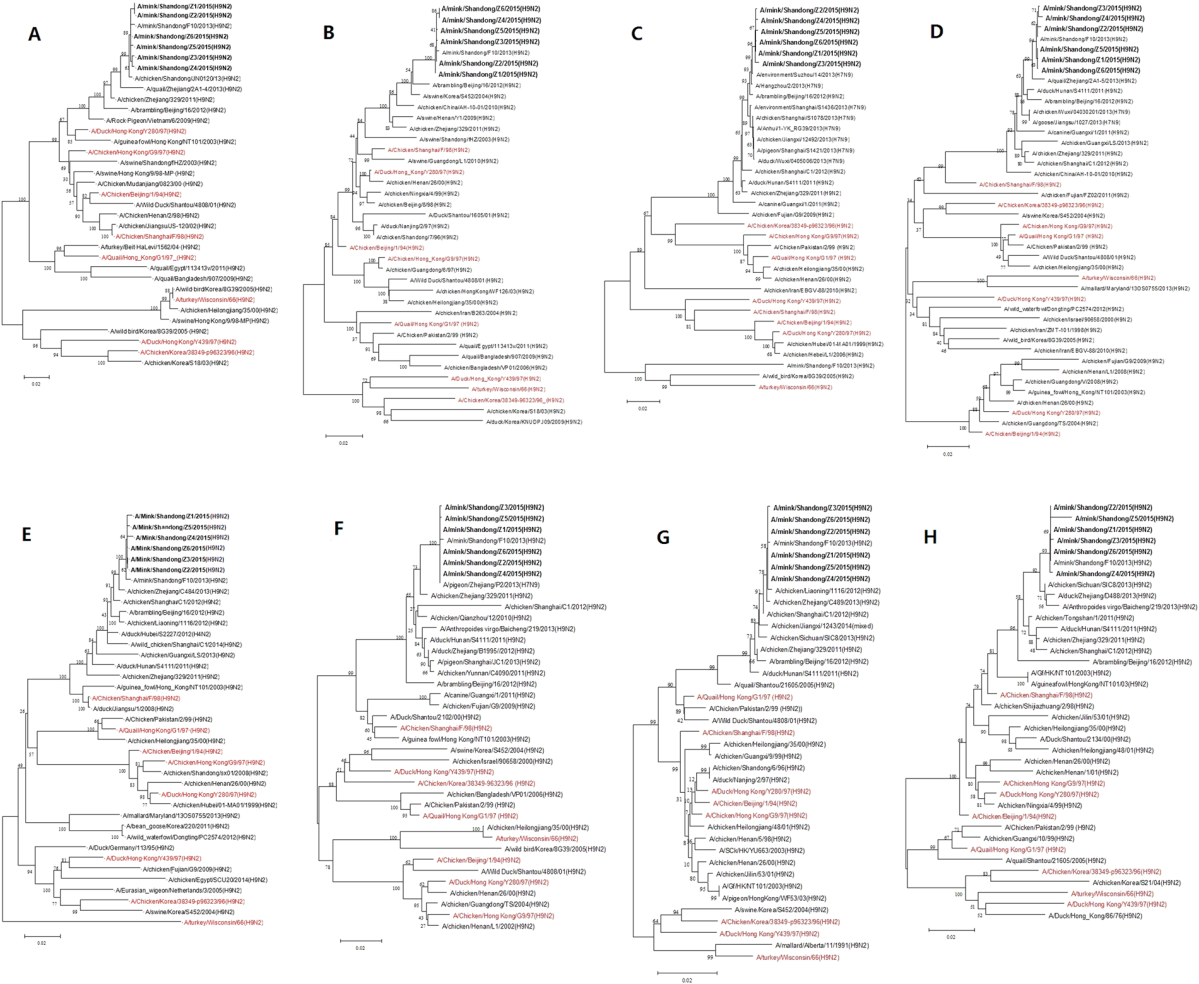
Phylogenetic trees of all eight segments of H9N2 IAVs isolated from the mink. Phylogenetic trees were constructed using MEGA 6.0, and the reliability of the tree was evaluated by the bootstrap method with 1,000 replications. The black bold sequences represented the H9N2 IAVs isolated from the mink, the red sequences represented the reference strains. (**A**), HA; (**B**), NA; (**C**), PB2; (**D**), PB1; (**E**), PA; (**F**), NP; (**G**), M; (**H**), NS.

The HA sequences showed 99.5–100% identity among the six isolates, NA 99.7–100%, PB2 99.5–99.9%, PB1 99.6–99.9%, PA 99.4–100%, NP 99.8–100%, M 99.7–100% and NS 99.2–100%. The similarity of the HA genes of the six isolates with the reference sequences were 79.2–97.7%, NA 79.4–98.5%, PB2 82.7–99.4%, PB1 85.3–99.6%, PA 84.6–99.7%, NP 87.7–99.9%, M 87.6–99.9% and NS 85.5–97.0%. The HA, NA, PB1, PA, NP, M and NS genes of the six isolates shared the highest nucleotide sequence identity with Mk/SD/F10/13, with a homology rate ranging from 99.2–100%. However, the PB2 genes of the six isolates shared the highest nucleotide sequence identity with A/environment/Suzhou/14/2013(H7N9), with a homology rate ranging from 99.2–99.4%. The phylogenetic trees were constructed using the nucleotide sequences of the six isolates and the corresponding genes of the reference viruses (Fig. 1). Phylogenetic analysis of HA genes revealed that the six isolates were similar to Y280-like viruses, indicating the six isolates belonged to the Eurasian lineage III. Phylogenetic analysis of the NA, PB1, PA, NP and NS genes showed that the six isolates clustered with Shanghai/F/98-like viruses. However, the M genes of the six isolates fell into G1-like lineage. The PB2 genes of the six isolates had a close relationship with the genes of A/environment/Suzhou/14/2013(H7N9), falling into Korean-like lineage.

The HA proteins of the six isolates contained an amino acid motif PSRSSR↓GL at their HA cleavage sites. Analysis of the potential HA N-glycosylation sites showed that eight sites with the N-X-T/S motif were conserved in the HA domain of the six isolates, six sites in the HA1 domain and two sites in the HA2 domain, 29–31 (NST), 82–84 (NPS), 141–143 (NVS), 298–300 (NTT), 305–307 (NVS), 313–315 (NCS), 492–494 (NGT) and 551–553 (NGS), which were identical to those of Mk/SD/F10/13. Compared with Y280, the six isolates lost the glycosylation site NRT at positions 218–220 and had the new glycosylation site NCS at positions 313–315 and site NGS at positions 551–553. The HA proteins of the six isolates encoded for L at position 234 (226 numbered according to H3). Analysis of the potential NA N-glycosylation sites of the six isolates showed that seven sites with the N-X-T/S motif were conserved in the NA proteins, which were identical to those of Mk/SD/F10/13 at positions 44–46 (NPS), 69–71 (NST), 86–88 (NWS), 146–148 (NGT), 200–202 (NAT), 234–236 (NGT), 264–266 (NIS). Because of the N296K mutation, the NA proteins of the six isolates lost a potential glycosylation site at position 296–298. Compared with the reference strains, the N61S mutation and 63–65 (TEI) deletion of the six isolates resulted in the deletion of the potential glycosylation site at position 61. The K368S and N402S mutations were observed at hemadsorbing (HB) sites in the NA proteins of the six isolates. The PB2 proteins of the six isolates contained 627E and 701 N.

### Experimental transmission

**Figure 2:**
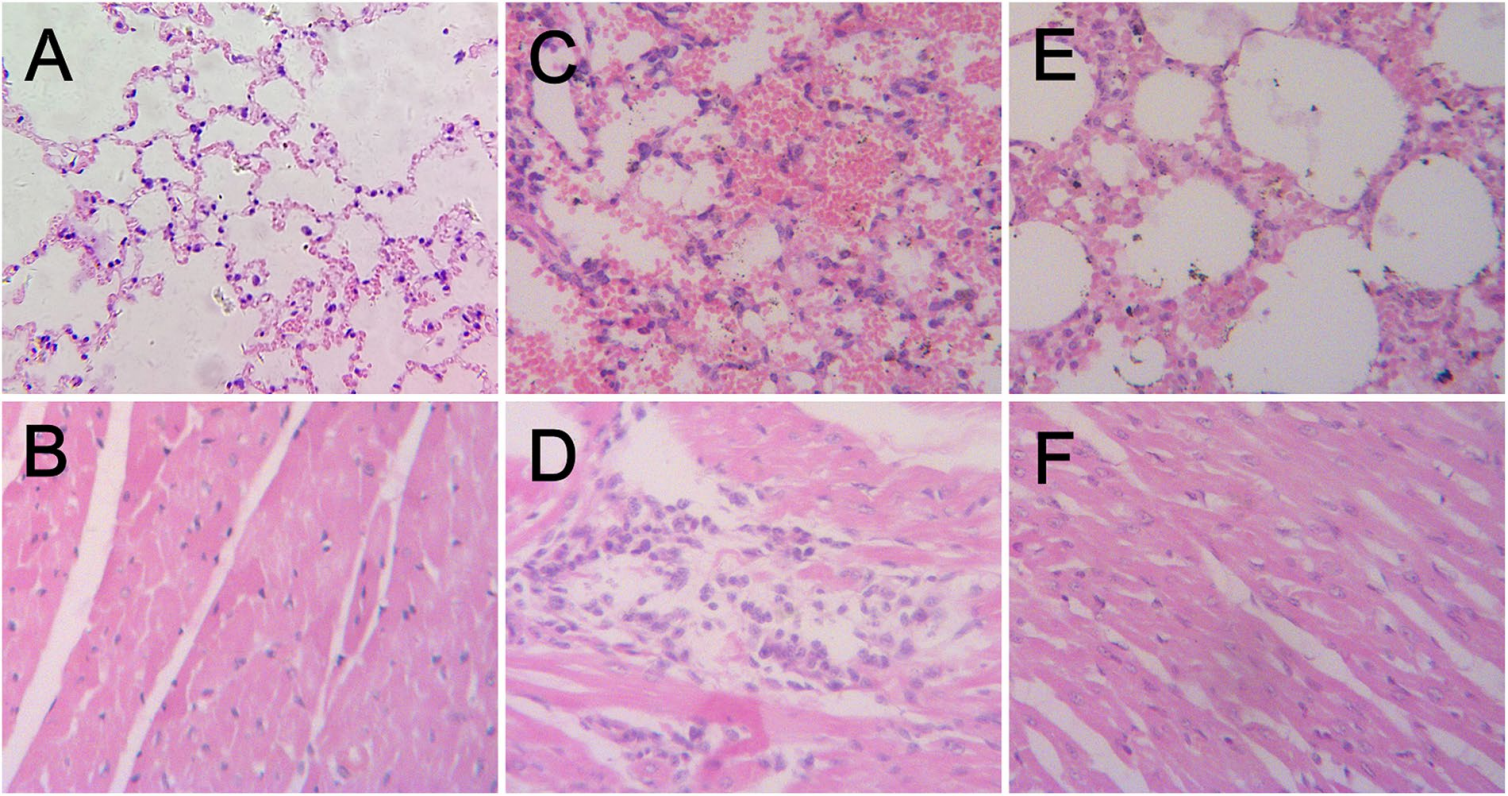
Histopathologic appearance of tissues of the mink. (**A**) Lung tissue taken from a control mink on day 6 p.i. (**B**) Heart tissue taken from a control mink on day 6 p.i. (**C**) Lung tissue taken from a inoculated mink on day 6 p.i., characterized by large area of bleeding, thickening of the alveolar septa. (**D**) Heart tissue taken from a inoculated mink on day 6 p.i., characterized by infiltration of inflammatory cells. (**E**) Lung tissue taken from an exposure mink on day 6 p.i., characterized by slight thickening of the alveolar septa. (**F**) Heart tissue taken from an exposure mink on day 6 p.i., no obvious histologic lesions were found. HE stain. Original magnification was ×200 for all images.

The donor and contact mink showed clinical signs, including loss of appetite, dullness, lethargy, mild coughing and sneezing on day 4–8 post infection (p.i.). The virus was detected in the nose swab sample of the contact mink on day 4 and 6 p.i., but not on day 8 p.i., and the titers of the nasal swab elutes were 10^3.1^–10^4.2^ EID_50_/mL. Obvious histologic lesions, such as large area of bleeding in lung, thickening of the alveolar septa and infiltration of inflammatory cells in heart were found in the donor mink euthanized on day 6 p.i., however, slight thickening of the alveolar septa was found in exposure mink euthanized on day 6 p.i. (Fig. 2). Trachea and lung tissues from the inoculated mink were positive for IAV by RT-PCR; only trachea tissues from the contact mink were positive. Moreover, seroconversion was detected in the inoculated and contact mink, and the HI titers of anti-H9 antibody were 128–1024 on day 14 p.i., without anti-H1 antibody. Finally, the mink recovered from disease. The contact foxes and raccoon dogs showed no clinic signs. The nasal and rectal swab samples of the contact foxes and raccoon dogs were negative for IAV by RT-PCR. Slight thickening of the alveolar septa was observed in the exposure fox and raccoon dog euthanized on day 8 p.i. All the tissue samples from the exposure foxes and raccoon dogs were negative for IAV. Finally, seroconversion was detected in all foxes and raccoon dogs, with HI titers of anti-H9 antibody ranging from 64 to 512.

### Pathogenesis experiments in foxes and raccoon dogs

The inoculated foxes and raccoon dogs showed no clinical signs. The nasal and rectal swab samples of the inoculated foxes and raccoon dogs were negative for IAV using RT-PCR. Histologic lesions were observed in the inoculated foxes and raccoon dogs euthanized on day 6 p.i., including slight thickening of the alveolar septa and infiltration of inflammatory cells in lung. All the tissue samples from the inoculated foxes and raccoon dogs were negative for IAV. Finally, seroconversion was detected in all foxes and raccoon dogs, with the HI titers of anti-H9 antibody ranging from 64 to 256.

## Discussion

H9N2 IAVs circulate widely in both wild birds and domestic poultry populations, and have transmitted to mammals such as swine and dogs^13, 18^, and even human^19–21^. Thirty one percent of mink serum samples were positive for anti-H9 antibody in 2015, higher than the 20% reported in 2013^5^, implying that H9N2 IAV had spread more widely in mink populations.

Phylogenetic analysis of the six isolates analyzed in our study showed that the HA, NA, PB1, PA, NP M and NS genes had a close relationship with the genes of Mk/SD/F10/13. The PB2 genes shared the highest nucleotide sequence identity with the PB2 gene of A/Hangzhou/2/2013(H7N9). The six isolates contained the amino acid motif PSRSSR↓GL at their HA cleavage sites, which is a characteristic of low pathogenic influenza viruses. There were two additional glycosylation sites at amino acid positions 313 and 551 of the HA proteins of the six isolates, but one glycosylation site was lost at amino acid position 218. Such changes of glycosylation in HA proteins likely contribute to the host specificity, virulence and infectivity^22^. The position 234 (226 numbered according to H3) in the receptor binding site of HA proteins of the six isolates encoded for L, which is a receptor characteristics of human influenza virus. The mutation might promote human infection with these^23^.

The N61S mutation and 63–65(TEI) deletion in the NA’s of the six isolates resulted in the deletion of a potential glycosylation site. The six isolates also lost a potential glycosylation site at position 296. The changes could affect the host specificity, virulence and infectivity in mink. The HB sites in the NA proteins of the six isolates were under positive selection pressure for mutations (K368S and N402S), which results from a functional match between HA and NA^10^.

Residue 627 of PB2 is a key factor for host range, and human influenza viruses have K at this position, whereas the majority of AIVs have E^24^. The six isolates contained the avian-like signature 627E although they contained 701 N which has been shown able to compensate for the lack of 627 K, increased polymerase activity and enhancing the replication of some strains of IAVs in mammalian hosts^25, 26^.

Transmission experiments showed that close contact between H9N2 infected mink and naïve contact mink, foxes and raccoon dogs resulted in spread of the virus to the sentinel animals as determined by virus isolation and/or seroconversion. H9N2-challenged foxes and raccoon dogs also showed H9N2 IAV could infect these animals without clinical signs and virus shedding, but with seroconversion. A serosurvey in foxes and raccoon dogs demonstrated that H9N2 IAV circulated in these hosts. In some areas in China, mink, foxes and raccoon dogs are raised on the same farms, which could increase the chance for H9N2 IAV to cross the species barrier. Our findings suggest that the potential exists for H9N2 IAV transmission to humans exposed to fur animals. Virological and epidemiological surveillance of IAVs in mink, foxes and raccoon dogs should be strengthened for public health.

## Methods

### Virus isolation

A total of 157 lung tissue samples (105 mink, 32 foxes and 20 raccoon dogs) were collected from euthanized farmed mink, foxes and raccoon dogs with ketamine chloride experiencing mild respiratory distress in Shandong, China, 2015. No poultry were housed on the farms. The mink, foxes and raccoon dogs inhabiting in the farms were fed with a ration composed of uncooked meat by-products of poultry. All samples were tested by RT-PCR based on IAV M gene. Positive samples were further titrated in 10-day-old specific-pathogen-free (SPF) embryonated chicken eggs, respectively. Virus isolation was performed as described previously^5^. In brief, the lung tissues were homogenized in 0.9% NaCl solution supplemented with 2000 units/ml penicillin, and 2000 mg/ml streptomycin, immediately centrifuged at 5000 × g for 5 min to precipitate debris. Subsequently, lung suspensions were inoculated into the allantoic cavities of 10-day-old SPF embryonated hens’ eggs. Eggs were incubated at 37 °C for 72 h, chilled at 4 °C overnight, and allantoic fluids were harvested and stored at −80 °C until analysis.

### Serosurvey of antibodies to H9N2 in mink, foxes and raccoon dogs

In 2015, 313 mink serum samples, 128 fox serum samples and 256 raccoon dogs serum samples were collected from 7 mink farms, 4 fox farms, and 5 raccoon dog farms, respectively, which were all unvaccinated with IAV vaccine. No poultry were housed on the farms. Serum samples were stored at −80 °C until analysis. The sera were adsorbed with chicken red blood cells to remove non-specific inhibitors of agglutination, and hemagglutination inhibition tests (HI) was performed using Mk/SD/F10/13(H9N2) and A/Swine/Shandong/JT/2007(H1N1), according to World Health Organization manual on animal influenza diagnosis and surveillance. HI titer of the serum is assigned as the reciprocal of the highest serum dilution by the viruses. The cut-off value of HI test was set as 16^27^.

### Gene sequence and molecular analysis

Total RNA was extracted from allantoic fluid with Transzol Up Reagent (Transgen, Beijing, China). The HA, NA, M, NP, NS, PB1, PB2, PA gene segments of the six isolates were obtained by RT-PCR using LA PCR Kit (AMV) Ver.1.1 (TaKaRa, Dalian, China) and the primers for the 8 genes of the IAV as described previously^5^ (Table 1). The PCR conditions were available upon request. RT-PCR products were extracted from agarose gels using an agarose gel DNA extraction kit (TaKaRa, Dalian, China), and sequencing was performed by Shanghai Sangon Biological. The nucleotide sequences of each gene of A/Mink/Shandong/Z1/2015, A/Mink/Shandong/Z2/2015, A/Mink/Shandong/Z3/2015, A/Mink/Shandong/Z4/2015, A/Mink/Shandong/Z5/2015, and A/Mink/Shandong/Z6/2015 were submitted to the GenBank, and assigned GenBank accession numbers KY272068 to KY272115.

**Table 1:** Primers used for RT-PCR.

GS	Type	Primers (5′–3′)	RV	GBA
PB1	F	aaagcaggcaaaccatttg	A/swine/Korea/S452/2004(H9N2)	AY790312.1
	R	ggcattttttcatgaaggaca		
PB2	F	aaagcaggtcaattatattc	A/chicken/Shandong/B1/1998(H9N2)	EU914196.1
	R	agtagaaacaaggtcgtttttaaac		
PA	F	aaagcaggtactgatcc	A/chicken/Guangdong/TS/2004(H9N2)	JQ639777.1
	R	agtagaaacaaggtacttttttg		
HA	F	agcaaaagcaggggaatttcac	A/chicken/Shaanxi/xy1/2012(H9N2)	KF638575.1
	R	agtagaaacaagggtgtttttgcc		
NP	F	atggcgtctcaaggcaccaaa	A/swine/Korea/S452/2004(H9N2)	AY790308.1
	R	tctttaattgtcatactcctctgca		
NA	F	agcaaaagcaggagtaaaaatg	A/chicken/Shandong/B4/2007(H9N2)	EU346938.1
	R	caaggagtttttttttaaaattgc		
M	F	gcaggtagatatttaaagatgagtc	A/swine/Korea/S452/2004(H9N2)	AY790306.1
	R	acaaggtagttttttactccagttc		
NS	F	agcgaaagcagggtgacaa	A/swine/Korea/S452/2004(H9N2)	AY790309.1
	R	tagaaacaagggtgttttttatca		

GS, Gene segment; RV, Reference viruses; GBA, GenBank accession no.

To further investigate the genotype and genetic origin of the isolates, the DNA sequences were compiled and edited using the Lasergene sequence analysis software package (DNASTAR, Madison, WI, USA). BLAST analyses (http://blast.ncbi.nlm.nih.gov/Blast.cgi) were used on each sequence to identify related reference viruses, and the nucleotide sequences of the related reference viruses were obtained from the GenBank database. The deduced amino acid sequences were compared with MEGA6.0 using Clustal W. Phylogenetic trees were constructed using MEGA6.0 by the neighbor-joining method. Bootstrap values were calculated on 1000 replicates of the alignment.

### Experimental transmission

To clarify whether the H9N2 IAV can be transmitted from mink to mink, foxes and raccoon dogs, the experimental groups of mink, foxes and raccoon dogs subdivided in 3 different groups were housed in separated isolation facility in different rooms, a total of 15 farmed mink (3–5 months of age) in the first group, 9 farmed mink (3–5 months of age) and 9 farmed foxes (3–4 months of age) in the second group, and 9 farmed mink (3–5 months of age) and 9 farmed raccoon dogs (3–4 months of age) in the third group. All the animals were negative for IAV antigen and anti-IAV antibody.

Among these three groups, 6 mink were lightly anesthetized with ketamine chloride and inoculated intranasally with a 10^5.0^ EID_50_ of virus in 0.5 ml PBS, using Mk/SD/F10/13 respectively. In the first group, the other 6 mink, serving as sentinel animals, were housed in adjacent cages in the same containment room to test for mink-to-mink intraspecies transmission of virus from day 1 p.i. In the second group, 6 foxes were housed in adjacent cages in the same containment room to test for mink-to-fox interspecies transmission of virus from day 1 p.i. In the third group, 6 raccoon dogs were housed in adjacent cages in the same containment room to test for mink-to-raccoon dog interspecies transmission of virus from day 1 p.i. Among the three groups, three mink, three foxes and three raccoon dogs were housed in a separate room, serving as negative controls. Clinical signs of infection were daily monitored p.i. To determine virus shedding, nasal and rectal swabs were collected from the animals on days 2 (donors only), 4, 6 and 8 p.i. The samples of swab elutes were firstly detected by RT-PCR based on HA, NA and M genes. If positive, the swab elutes were further titrated in 10-day-old SPF embryonated chicken eggs, respectively. Two sentinel mink, two sentinel foxes and two sentinel raccoon dogs from treatment groups and one mink, one fox, and one raccoon dog from control group were euthanized on days 6 and 8 p.i., respectively. The animals were necropsied and tissues were taken for histopathology and routine virologic examinations, including lung, trachea, cerebrum, cerebellum, brain stem, duodenum, stomach, colon, pancreas, spleen, liver, kidney, urinary bladder, lymph node and heart. A part of the samples were rapidly immersed in 10% neutral formalin buffer to prevent autolysis, and then processed into paraffin, sectioned at 4 µm using the microtome Leica RM2235 (Leica Microsystems Ltd.), and stained with hematoxylin-eosin (HE) for the identification of pathological features by light microscopy. The other samples were stored at −80 °C until for virological examinations by RT-PCR. Serum samples were collected on 14 day p.i. for serological testing using HI.

### Pathogenesis experiments in foxes

To determine the pathogenicity of H9N2 IAV for foxes, experiments were performed on 10 3–4 months old farmed foxes, which were negative for IAV antigen and anti-IAV antibody. Ten foxes were divided into 2 groups. The 5 foxes of the first group were lightly anesthetized with ketamine chloride and inoculated intranasally with a 10^5.0^ EID_50_ of virus in 0.5 ml PBS, using Mk/SD/F10/13. The 5 foxes of the second group were inoculated intranasally with 0.9% NaCl solution, serving as negative controls. All foxes in both groups were kept in separated rooms of the isolation facility. From day 1 p.i. onwards, clinical signs of the foxes were monitored. Nasal and rectal swabs were collected on days 2, 4, 6 and 8 p.i and screened for the virus shedding. The swab elutes were tested as above. One inoculated fox and one control fox were euthanized on days 4, 6 and 8 p.i. respectively. The tissue samples were collected and tested as above. Serum samples were collected at 14 days p.i. for serological testing using HI.

### Pathogenesis experiments in raccoon dogs

To determine the pathogenicity of H9N2 IAV for raccoon dogs, experiments were performed on 10 3–4 months old farmed raccoon dogs, which were negative for IAV antigen and anti-IAV antibody. Ten raccoon dogs were divided into 2 groups. The 5 raccoon dogs of the first group were lightly anesthetized with ketamine chloride and inoculated intranasally with a 10^5.0^ EID_50_ of virus in 0.5 ml PBS, using Mk/SD/F10/13. The 5 raccoon dogs of the second group were inoculated intranasally with 0.9% NaCl solution, serving as negative controls. All raccoon dogs in both groups were kept in separated rooms of the isolation facility. Clinical signs of the raccoon dogs were monitored daily from day 1 p.i. onwards. To determine virus shedding, nasal and rectal swabs were collected from the animals on days 2, 4, 6 and 8 p.i. The swab elutes were detected as above. One inoculated raccoon dog and one control raccoon dogs were euthanized on days 4, 6 and 8 p.i., respectively. The tissue samples were collected and tested as above. Serum samples were collected at 14 days p.i. for serological testing using HI.

### Ethics statement

This study was carried out according to the current laws of China, and also complied with the European Union Animal Welfare legislation. The study was performed in Biosafety Level 2 containment. All animal experiments were performed in accordance with regulatory standards and guidelines approved by the Shandong Agricultural University’s Animal Care and Use Committee, and the approved NO. is SDAUA-2015-009.
